# PICT-1 is a key nucleolar sensor in DNA damage response signaling that regulates apoptosis through the RPL11-MDM2-p53 pathway

**DOI:** 10.18632/oncotarget.13082

**Published:** 2016-11-04

**Authors:** Hongbo Chen, Liqiao Han, Hsiangi Tsai, Zhiwei Wang, Yanping Wu, Yanhong Duo, Wei Cao, Lijun Chen, Zhirong Tan, Ning Xu, Xianzhang Huang, Junhua Zhuang, Laiqiang Huang

**Affiliations:** ^1^ The Shenzhen Key Lab of Gene and Antibody Therapy, Center for Biotechnology & Biomedicine, Division of Life and Health Sciences, Graduate School at Shenzhen, Tsinghua University, Shenzhen 518055, China; ^2^ School of Life Sciences, Tsinghua University, Beijing 100084, China; ^3^ Department of Laboratory Science, The Second Affiliated Hospital of Guangzhou University of Chinese Medicine, Guangzhou 510120, China; ^4^ Department of Laboratory Medicine, The Fourth Affiliated Hospital of Guangzhou Medical University, Guangzhou 511447, China; ^5^ Technology Center of Guangxi Entry-Exit Inspection and Quarantine Bureau, Nanning 530021, China; ^6^ Department of Biochemistry, McGill University, Montreal, QC H3G 1Y6, Canada

**Keywords:** PICT-1, nucleolus, DNA damage, nucleolar stress

## Abstract

PICT-1 is an essential ribosome biogenesis factor whose loss induces p53 accumulation and apoptosis. Here, we show that DNA damage changes PICT-1 localization and decreases PICT-1 protein levels via the proteasome pathway. Two important phosphatidylinositol 3-kinase-like kinases (PIKKs), ataxia-telangiectasia mutated (ATM) and the Ku70 subunit of DNA-dependent protein kinase (DNA-PK), co-localize and interact with PICT-1 in the nucleolus. Computational prediction of phosphorylation sites and detection using an anti-phospho-substrate antibody suggest that PICT-1 might be a substrate of PIKKs. PICT-1 S233 and T289 were identified as the key phosphorylation sites in this pathway, as mutating both to alanine abolished UVB-induced increase of PICT-1 phosporylation. Inhibition of PIKKs or ATM (with wortmannin and KU55933, respectively) prevented the agglomeration and degradation of PICT-1, suggesting that ATM is a key regulator of PICT-1. PICT-1(S233A, T289A) demonstrated marked resistance to DNA damage-induced agglomeration and loss of PICT-1. Phosphomimetic PICT-1 (S233D, T289D) showed a different nuclear distribution and was more rapidly degraded after DNA damage than wild-type PICT-1. Furthermore, both phosphorylation and degradation of PICT-1 released RPL11 from the nucleolus to the nucleoplasm, increased binding of RPL11 to MDM2, and promoted p53 accumulation and apoptosis in an ATM-dependent manner after DNA damage. These data indicate that PICT-1 is a major nucleolar sensor of the DNA damage repair response and an important upstream regulator of p53 via the RPL11-MDM2-p53 pathway.

## INTRODUCTION

The protein interacting with carboxyl terminus 1 (PICT-1) is encoded by glioma tumor suppressor candidate region gene 2 (GLTSCR2), located at human chromosome 19q13.32 [1, 2]. PICT-1 is frequently lost in gliomas and was therefore identified as a candidate tumor suppressor [2]. In support of this role, PICT-1 expression is negatively correlated with the development and progression of ovarian cancer and glioblastomas [3–6]. PICT-1 achieves this function by directly interacting with and stabilizing the tumor suppressor phosphatase and tensin homolog protein (PTEN) in the cytoplasm, thereby inhibiting the PI3K/PIP3 pathway [7, 8]. Accordingly, in HEK293 and glioma cells, PICT-1 knockdown promotes cell proliferation and decreases susceptibility to apoptosis in a PTEN-dependent manner. In contrast, PICT-1 overexpression inhibits growth and proliferation, and promotes apoptosis [7–9]. Although many studies support a role for PICT-1 as a tumor suppressor, some contradictory findings have been reported. For example, in patients with anaplastic oligodendrogliomas, loss of heterozygosity at chromosome 19q13 or deletion of the chromosome 19q arm lengthens survival after chemotherapy [1, 10, 11]. Therefore, further studies are needed to elucidate the function of PICT-1 in normal and cancerous cells.

PICT-1 preferentially localizes to nucleoli [12–15]. The nucleolus has traditionally been thought of as the site of ribosome biogenesis [16]. However, recent evidence suggests that the nucleolus also participates in diverse biological processes, including the cell cycle, aging, development, and apoptosis [17–21]. For example, the nucleolus can control cell death by regulating the murine double minute 2 (MDM2)/p53 signaling pathway [22–25]. Normally, MDM2 binds p53 and promotes its ubiquitination and proteasomal degradation in the nucleoplasm. Under conditions of cell stress, nucleolar disruption occurs and several ribosomal proteins (RPs) (including S7, L5, L11, L23 and L26) are translocated from the nucleolus to the nucleoplasm. These RPs bind MDM2 and inhibit its ubiquitin ligase function, thereby allowing p53 activity [22–26]. While several newly synthesized RPs are degraded by proteasomes under ribosomal biogenesis stress, RPs L5 and L11 are protected from proteasomal degradation and selectively accumulate in the ribosome-free fraction, indicating they may be particularly important for p53 accumulation [27]. 5S rRNAs also play key role in regulating the response to nucleolar stress. Depletion of any one of the three components of 5S ribonucleoprotein (5S RNP) (5S rRNA, RPL5, or RPL11) abolished binding of the other two to MDM2, and blocks stress-induced p53 activation [28, 29]. More importantly, PICT-1 can directly bind 5S rRNA and is responsible for 5S RNP integration into the ribosome, making it an essential ribosome biogenesis factor [28]. In normal cells, PICT1 retains RPL5 and RPL11 in the nucleolus by directly binding 5S RNP, and PICT-1 depletion increases RPL11 translocation to the nucleoplasm and p53 activation [28, 30–32].

Many environmental factors, such as ionizing radiation, UV radiation, and chemical mutagens, can damage DNA. In response to DNA damage, the DNA damage response (DDR) signaling pathway in eukaryotic cells sequentially recognizes damage sites, activates checkpoint proteins to delay cell cycle progression, and recruits DNA repair proteins [33–35]. PICT-1 down-regulation sensitizes cells to DNA damage, indicating that PICT-1 might participate in the cross-talk between DDR signaling and the nucleolar stress response [36, 37]. However, the exact molecular mechanisms underlying this process are unknown.

Here, we find that PICT-1 was a substrate of ATM in the nucleolus. DNA damage changed the distribution and down-regulated levels of PICT-1 protein in an ATM-dependent manner. Accordingly, DNA damage-induced phosphorylation and degradation of PICT-1 resulted in the release of RPL11 from nucleoli to nucleoplasm, p53 accumulation, and apoptosis. Thus, our evidence suggests that PICT-1 links DDR signaling and the nucleolar stress response.

## RESULTS

### DNA damage alters the distribution pattern and decreases levels of PICT-1 protein via a proteasome-dependent pathway

**Figure 1:**
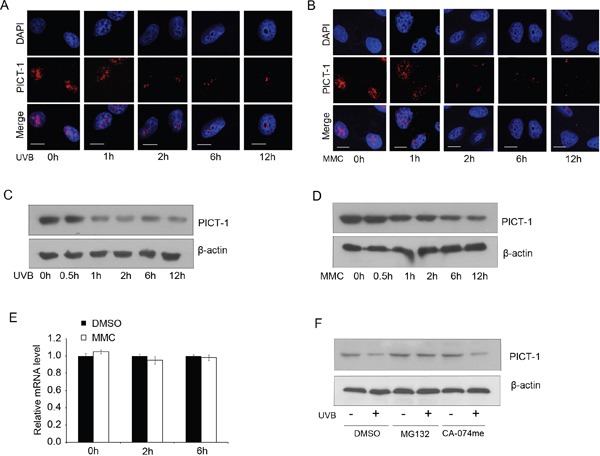
UVB radiation and MMC treatment alter PICT-1 distribution and protein levels **A-B.** HEK293 cells were exposed to UVB radiation (10 J/m^2^) (A) or MMC (10 μg/mL) (B), then PICT-1 was immunostained and detected using confocal microscopy. PICT-1: red; DAPI: blue; Scale bar = 10 μm. **C-D.** HEK293 cells were exposed to UVB radiation (10 J/m^2^) (C) or MMC (10 μg/mL) (D) and PICT-1 was detected by western blotting at the indicated time points. **E.** HEK293 cells were exposed to UVB radiation (10 J/m^2^) and PICT-1 mRNA was quantified using qRT-PCR at the indicated time points. Results are presented as mean ± SD from three independent experiments. **F.** Naive or pre-treated (10 μM MG132 or 20 μM CA-074Me, 10 min) cells were exposed to UVB light. 6 h after UVB exposure, cell lysates were analyzed by western blotting using indicated antibodies.

DNA damage can cause mutagenesis and contribute to cancer development. To prevent these events, cells have evolved the DDR pathway, which consists of many proteins that change in abundance and localization during DDR. To investigate the effects of DNA damage on PICT-1, we induced DNA damage in HEK293 cells with UVB light (10 J/m^2^) or the DNA-damaging chemical mitomycin C (MMC, 10 μg/mL). We then studied the subcellular distribution of PICT-1 by confocal microscopy. Consistent with previous reports [15], PICT-1 protein in untreated cells showed diffuse staining accompanied by dispersed puncta throughout the nucleolus. In contrast, diffuse PICT-1 staining significantly decreased early after UVB radiation (Figure 1A) or MMC exposure (Figure 1B). Dispersed puncta then gradually coalesced into larger puncta finally forming one or two large puncta in the nucleolus 12 h after DNA damage (Figure 1A, 1B). To evaluate overall levels of PICT-1, HEK293 were exposed to UVB light or MMC, and immunoblot analysis was performed on cell lysates at different post-treatment time points. As shown in Figure 1C and 1D, PICT-1 levels gradually decrease after UVB or MMC exposure. Similar results were obtained from U251 cells (data not shown). These data demonstrate that DNA damage agents decrease PICT-1 levels while causing aggregation of the remaining PICT-1 protein.

To investigate the mechanism of PICT-1 downregulation, we next performed quantitative real-time PCR (qRT-PCR) for PICT-1 mRNA following UVB treatment. PICT-1 mRNA levels were unaffected by UVB exposure for the first 6 h after radiation (Figure 1E). This suggests that the UVB-mediated decrease in PICT-1 protein levels is not due to transcriptional repression. Protein degradation in mammalian cells is primarily mediated by either the proteasome or lysosome [38, 39]. More specifically, Maehama *et al* reported thatnucleolar stress induced by actinomycin D, doxorubicin, or FUrd caused significant downregulation of PICT-1 via proteasomal degradation [40]. Thus, we treated control or UVB-exposed cells with the proteasome inhibitor MG132 or the lysosome inhibitor CA-074me and measured PICT-1 protein. As expected, MG132, but not CA-074me, treatment significantly blocked UVB-induced PICT-1 degradation (Figure 1F). Taken together, our data show that DNA damage causes PICT-1 aggregation and proteasome-dependent degradation.

### PICT-1 is a substrate of PIKKs

**Figure 2:**
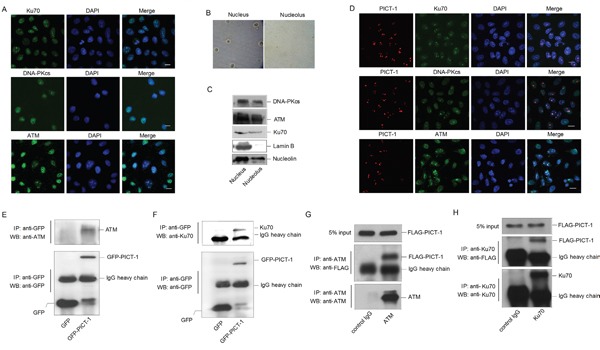
ATM and DNA-PK localize to nucleoli and interact with PICT-1 **A.** HEK293 cells were immunostained with anti-ATM, DNA-PKcs or Ku70 antibodies. Cells were imaged using confocal microscopy. Scale bar = 10 μm. **B.** HEK293 cells were fractionated into nuclear and nucleolar fractions. **C.** Lysates were analyzed by western blotting using the indicated antibodies. Lamin B was used as a nucleoplasmic marker. **D.** HEK293 cells were transfected with DsRedC1-PICT-1, then immunostained with anti-ATM, DNA-PKcs or Ku70 antibodies. Cells were imaged using confocal microscopy. Scale bar = 10 μm. **E-F.** HEK293 cells were transfected with pEGFPC1 or pEGFPC1-PICT-1 plasmids for 24 h. Cell lysates were then immunoprecipitated with an anti-GFP antibody and subjected to western blot analysis with anti-ATM or anti-Ku70 antibodies, respectively. **G-H.** HEK293 cells were transfected with pFLAG-CMV2-PICT-1 for 24 h. Cell lysates were then immunoprecipitated with anti-ATM or anti-Ku70 antibodies and subjected to western blot analysis with FLAG antibody.

Ataxia-telangiectasia mutated (ATM) and DNA-dependent protein kinase (DNA-PK) are members of phosphatidylinositol 3-kinase-like kinases family (PIKKs), which are rapidly activated in response to DNA damage [33]. Activated PIKKs phosphorylate a series of proteins to cause cell cycle arrest and DNA damage repair. Interestingly, the online software Group-Based Prediction System (GPS 2.1, http://gps.biocuckoo.org/) predicts that PICT-1 residues S233 and T289 may be phosphorylated by ATM and DNA-PK (Table 1). However, it is unknown whether ATM and DNA-PK localize to the nucleolus. To investigate this question, a modified immunocytochemical assay was performed according to previous report [42] in HEK293 cells. As shown in Figure 2A, ATM, DNA-PKcs (the catalytic subunit of DNA-PK) and Ku70 (a regulatory subunit of DNA-PK) were clearly localized to the nucleoli, in addition to showing diffuse staining throughout the nucleoplasm. Furthermore, subcellular fractionation was performed to isolate nuclear and nucleolar fractions for western blotting [41] (Figure 2B). As shown in Figure 2C, ATM, DNA-PKcs and Ku70 are present in the nucleolar fraction. To investigate whether PICT-1 co-localizes with these proteins, HEK293 cells were transfected with the DsRedc1-PICT-1 plasmid for 24 h. Immunocytochemical staining was performed with anti-ATM, anti-DNA-PKcs, or anti-Ku70 antibodies. ATM, DNA-PKcs, and Ku70 all co-localized with PICT-1 in nucleoli (Figure 2D). We next performed a co-immunoprecipitation (Co-IP) assay to determine whether PICT-1 interacts with ATM and DNA-PK. HEK293 cells were transfected with the pEGFPC1-PICT-1 plasmid for 24 h, and PICT-1 was pulled down using an anti-EGFP antibody. Western blots of the immunoprecipitate revealed that PICT-1 binds to ATM (Figure 2E) and Ku70 (Figure 2F) but not DNA-PKcs (data not shown). Furthermore, we performed the reciprocal Co-IPs using ATM and Ku70 antibodies. As shown in Figure 2G and 2H, PICT-1 was also specifically co-immunoprecipitated using ATM or Ku70 antibody, respectively. These data suggest that PICT-1 co-localizes with and binds ATM and DNA-PK in nucleoli.

**Table 1:** Prediction of PIKKs-specific phosphorylation sites by GPS 2.1 software

Position	Kinase	Peptide	GPS score	Cutoff score
233	ATM	TKPSQAP	4.023	2.6
233	DNA-PK	TKPSQAP	3.111	2.1
289	ATM	QAATQES	4.591	2.6
289	DNA-PK	QAATQES	3.278	2.1

**Figure 3:**
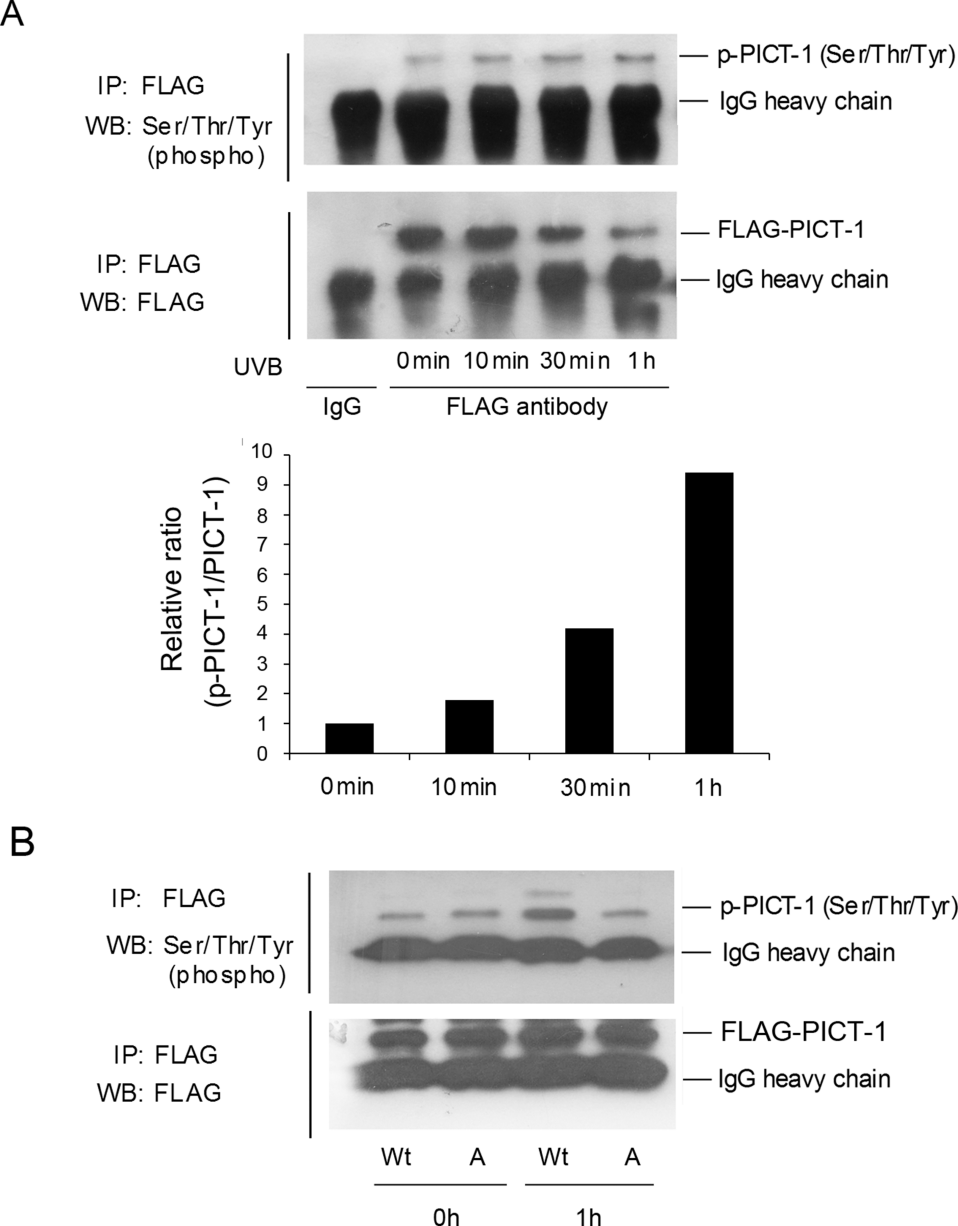
DNA damage induces PIKKs-dependent phosphorylation and degradation of PICT-1 **A.** HEK293 cells were transfected with pFLAG-CMV2-PICT-1 plasmid for 24 h, then UVB-irradiated (10 J/m^2^). FLAG-PICT-1 was immunoprecipitated with an anti-FLAG antibody at the indicated time points. Phosphorylated and total FLAG-PICT-1 was detected using anti-phospho-(Ser/Thr/Tyr) antibody or FLAG antibody. Relative band intensities were quantified by densitometry and the ratios of phosphorylated to total PICT-1 are shown. **B.** Cells were transfected with pFLAG-CMV2-PICT-1 or pFLAG-CMV2-PICT-1 (S233A, T289A) plasmids and exposed to UVB light for 1 h. FLAG-PICT-1 was immunoprecipitated and phosphorylated FLAG-PICT-1 was detected using anti-phospho-(Ser/Thr/Tyr) antibody. Wt: FLAG-PICT-1; A: FLAG-PICT-1 (S233A, T289A).

In order to determine whether PICT-1 is a substrate of DNA-PK or ATM, HEK293 cells were transfected with the pFLAG-CMV2-PICT-1 plasmid. Cells were exposed to UVB radiation (10 J/m^2^), and phosphorylation levels of FLAG-PICT-1 fused protein were then detected using the anti-phospho-(Ser/Thr/Tyr) antibody. As shown in Figure 3A, the ratio of phosphorylated to total FLAG-PICT-1 in UVB-irradiated cells significantly increased by 10 min and peaked at 1 h after UVB radiation. We then transfected HEK293 cells with wild-type or unphosphorylatable (S233A, T289A) PICT-1 for 24 h and exposed them to UVB radiation. PICT-1 phosphorylation was significantly lower in cells transfected with mutant than in wild-type PICT-1 1 h after UVB radiation (Figure 3B). These findings demonstrate that PICT-1 S233 and T289 are phosphorylated by PIKKs in response to DNA damage.

### DNA damage-induced changes in PICT-1 distribution and protein levels require ATM activation and PICT-1 phosphorylation

**Figure 4:**
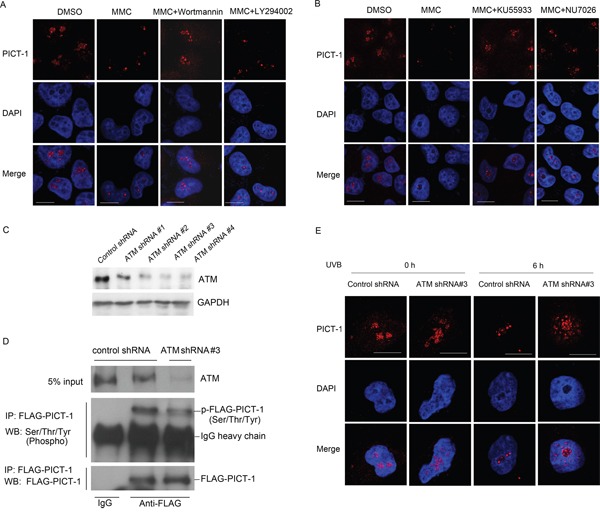
DNA damage-induced changes in distribution and expression of PICT-1 are dependent on ATM activation **A.** Naive or pre-treated (wortmannin or LY294002, 30 min) cells were incubated with MMC (10 μg/mL) for 6 h. Endogenous PICT-1 was detected using confocal microscopy. Scale bar = 10 μm. **B.** Naive or pre-treated (KU55933 or NU7026, 30 min) cells were incubated with MMC (10 μg/mL) for 6 h. Endogenous PICT-1 was detected using confocal microscopy. Scale bar = 10 μm. **C.** HEK293 cells were infected with ATM shRNA lentiviruses, and western blot was performed to detect the ATM expression. **D.** HEK293 cells infected with ATM shRNA#3 or control shRNA were transfected with pFLAG-CMV2-PICT-1 for 24h and subjected to UVB radiation, co-IP was performed using anti-FLAG antibody and phosphorylated PICT-1 was detected. **E.** HEK293 cells infected with ATM shRNA#3 or control shRNA were subjected to UVB radiation and endogenous PICT-1 was observed by confocal microscopy at 0 h and 6 h post-radiation. Scale bar = 10 μm.

The proteasomal degradation of some proteins can be promoted by phosphorylation [43]. To investigate the role of PIKKs in this phenomenon, HEK293 cells were pre-treated with wortmannin or LY294002 for 30 min and then treated with MMC for 6 h. Wortmannin is a broad inhibitor of PIKKs that irreversibly inhibits DNA-PK and ATM, whereas LY294002 is a potent inhibitor of class I phosphoinositide 3-kinases but does not efficiently inhibit PIKKs even at high concentrations [42, 44]. MMC treatment significantly increased ATM phosphorylation, and wortmannin, but not LY294002, attenuated this increase (data not shown). Similarly, wortmannin, but not LY294002, prevented MMC-induced changes in the cellular distribution of PICT-1 as measured by immunofluorescence staining (i.e. wortmannin-treated cells maintained diffuse PICT-1 staining after MMC treatment) (Figure 4A). These findings suggest that PICT-1 degradation and aggregation require activation of one or more PIKKs. To identify the kinase(s) responsible for these changes, cells were pre-treated with the ATM specific inhibitor KU55933 or the DNA-PK specific inhibitor NU7026, and then treated with MMC for 6 h. Both KU55933 and NU7026 potently attenuated the aggregation and degradation of PICT-1 (Figure 4B). However, KU55933 has a more significant effect than NU7026, suggesting that ATM is the key regulator of PICT-1 in response to DNA damage. To further confirm this, we performed a knockdown of ATM in HEK293 cells using several lentiviral based shRNAs and shRNA#3 was shown to have a high knockdown effect (Figure 4C). HEK293 cells infected with shRNA#3 were transfected with pFLAG-CMV2-PICT-1 were subjected to UVB treatment. Co-IP was performed using anti-FLAG antibody and phosphorylated PICT-1 was detected. As shown in Figure 4D, the phosphorylation level of PICT-1 in ATM knockdown cells is markedly lower than that in control cells following UVB treatment. Consistently, PICT-1 showed a obvious resistance in aggregation and degradation to UVB radiation in ATM knockdown cells compared to control cells (Figure 4E).

**Figure 5:**
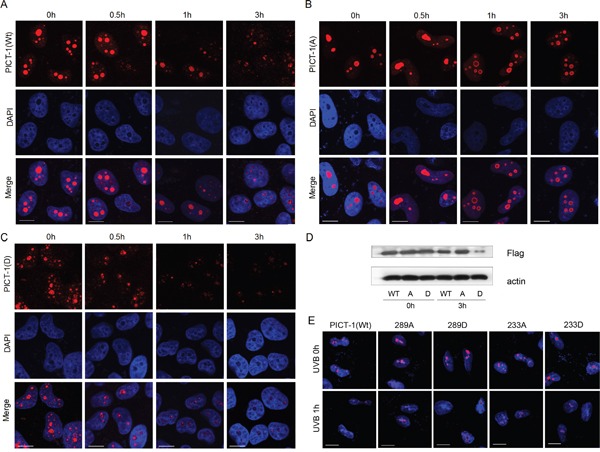
S233 and T289 phosphorylation affect amount and nucleolar distribution of PICT-1 protein in response to DNA damage **A-D.** HEK293 cells were transfected with pFLAG-CMV2-PICT-1, pFLAG-CMV2-PICT-1 (S233A, T289A) or pFLAG-CMV2-PICT-1 (S233D, T289D) for 24 h. Cells were then treated with MMC (10 μg/mL). A-C. FLAG-fused proteins were stained with anti-FLAG antibodies and detected using confocal microscopy. (D) FLAG-fused proteins were detected by western blotting 3 h post-MMC treatment. Wt: FLAG-PICT-1; A: FLAG-PICT-1 (S233A, T289A); D: FLAG-PICT-1 (S233D, T289D). **E.** HEK293 cells were transfected with pFLAG-CMV2-PICT-1, pFLAG-CMV2-PICT-1 (S233A), (S233D), (T289A) or (T289D) for 24 h. Cells were then treated with UVB, FLAG-fused proteins were stained with anti-FLAG antibodies and detected using confocal microscopy.

To investigate the effect of phosphorylation on the cellular distribution and expression of PICT-1, HEK293 cells were transfected with plasmids encoding FLAG-tagged wild-type, unphosphorylatable (S233A, T289A), or phosphomimetic (S233D, T289D) PICT-1 for 24 h and then exposed to MMC. Cells were immunostained with anti-FLAG antibody before visualization using confocal microscopy. MMC treatment reduced nucleolar fluorescence intensity in wild-type FLAG-PICT-1 transfected cells 1 h after MMC exposure (Figure 5A), reflecting MMC-induced PICT-1 degradation. Whereas, unphosphorylatable PICT-1 (S233A, T289A) showed obvious resistance to MMC-induced protein degradation in nucleoli (Figure 5B). Furthermore, MMC treatment decreased PICT-1 immunostaining more rapidly in phosphomimetic FLAG-PICT-1 than in wild-type transfected cells (Figure 5C). In addition, FLAG-PICT-1 (S233D, T289D) staining revealed puncta throughout the nucleus, contrasting with the nucleolar localization of wild-type FLAG-PICT-1. Western blotting confirmed that FLAG-PICT-1 (S233A, T289A) is resistant to MMC-induced protein degradation compared to wild-type and phosphomimetic FLAG-PICT-1 (Figure 5D). These data suggest that S233 and T289 phosphorylation controls the distribution and degradation of PICT-1 in response to DNA damage.

Furthermore, we tested the phosphorylation sites separately. As shown in Figure 5E, UVB treatment reduced nucleolar fluorescence intensity in wild-type FLAG-PICT-1 transfected cells 1 h after UVB exposure. Whereas, unphosphorylatable single site mutation (T289A but not S233A) showed a weak resistance to UVB-induced protein degradation in nucleoli (Figure 5E), suggesting that T289 site might be a important phosphorylation site that is responsible for DNA damage-induced degradation of PICT-1. In addition, single phosphomimetic site mutation (both S233D and T289D) did not showed a puncta pattern throughout the nucleus as FLAG-PICT-1 (S233D, T289D) (Figure 5E), which indicated that the phosphorylation in both sites has a more significant effect on DNA damage-induced PICT-1 degradation than single site phosphorylation.

### DNA damage-induced phosphorylation and degradation of PICT-1 regulates p53 accumulation via RPL11-MDM2

**Figure 6:**
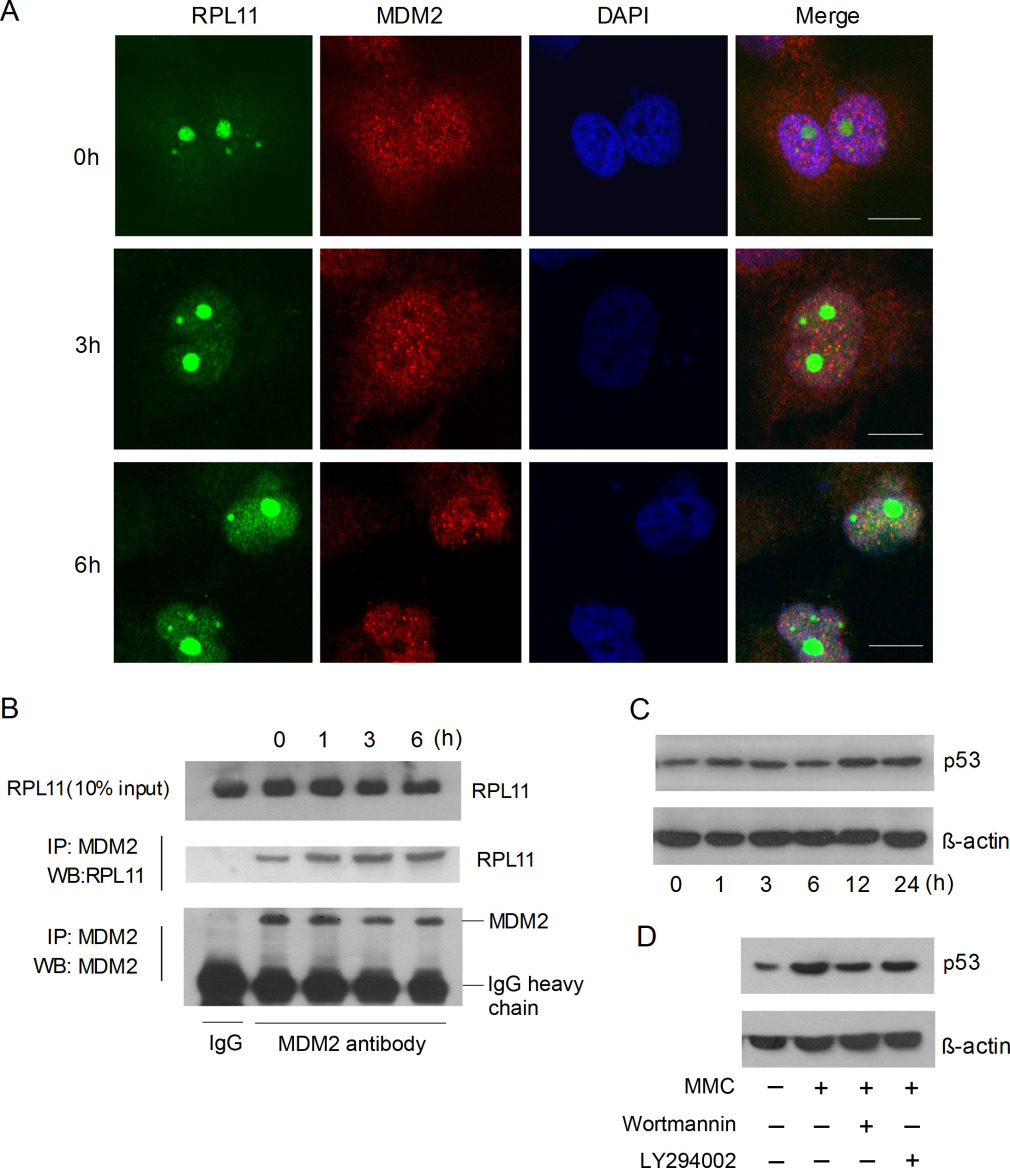
DNA damage induces p53 accumulation via the RPL11-MDM2 pathway **A.** HEK293 cells were transfected with pFLAG-CMV2-RPL11 for 24 h and treated with MMC (10 μg/mL). FLAG-RPL11 and MDM2 were detected with antibodies against FLAG (green) or MDM2 (red) by confocal microscopy at the indicated time points. Scale bar = 20 μm. **B.** U251 cells were treated with MMC (10 μg/mL). Cell lysates were immunoprecipitated using anti-MDM2 antibody and RPL11 was detected by western blot at the indicated time points. **C.** HEK293 cells were treated with MMC (10 μg/mL) and p53 were detected by western blot at the indicated time points. **D.** HEK293 cells were treated with MMC (10 μg/mL) in the presence or absence of wortmannin or LY294002 for 6 h, and p53 was then detected by western blot.

PICT-1 is an essential ribosome biogenesis factor responsible for 5S RNP integration into the ribosome. Depletion of PICT-1 relocates RPL11 to the nucleoplasm, where it binds to and blocks MDM2 activity, allowing p53 accumulation [28, 30]. Therefore, we investigated the effects of DNA damage incurred by UVB radiation on RPL11-MDM2 interaction and p53 levels in HEK293 cells. UVB radiation resulted in the transolcation of RPL11 from nucleolus to nucleoplasm and the co-localization of RPL11 and MDM2 (Figure 6A). Accordingly, UVB exposure significantly increased the binding of RPL11 and MDM2 proteins, determined by Co-IP (Figure 6B). As expected, p53 levels also significantly increased following UVB treatment (Figure 6C). To investigate the role of PIKKs in p53 accumulation, cells were pre-treated with Wortmannin or LY294002 for 30 min and then exposed to UVB light. wortmannin, but not LY294002, attenuated the UVB-induced increase of p53 (Figure 6D). These findings suggest that DNA damage-induced p53 accumulation depends in part on PIKKs-mediated PICT-1 degradation.

**Figure 7:**
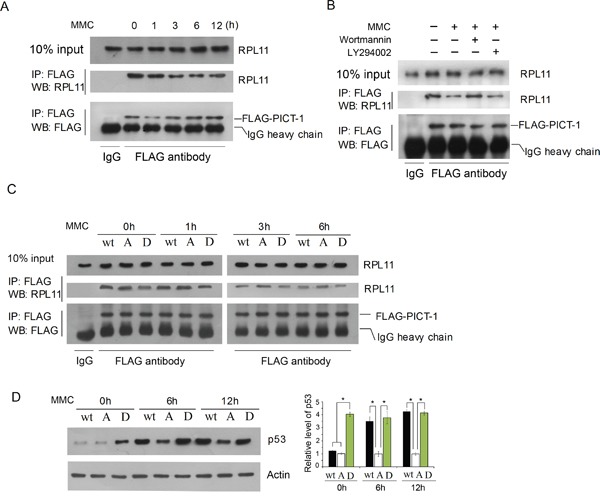
Phosphorylation of PICT-1 decreases its binding to RPL11 **A.** HEK293 cells were transfected with pFLAG-CMV2-PICT-1 for 24 h. Cells were then either left untreated or were treated with MMC (10 μg/mL). The same amounts of FLAG-PICT-1 protein were immunoprecipitated in both groups, and co-precipitated RPL11 protein was detected by western blotting. **B.** Cells were treated as in (A), but MMC treatment occurred in the presence or absence of wortmannin or LY294002 for 6 h. **C.** HEK293 cells were transfected with pFLAG-CMV2-PICT-1, pFLAG-CMV2-PICT-1 (S233A, T289A) or pFLAG-CMV2-PICT-1 (S233D, T289D) for 24 h. Cells were left untreated or were incubated with MMC (10 μg/mL). The same amounts of FLAG-PICT-1 protein were immnoprecipitated in each group, and co-precipitated RPL11 proteins were detected by western blotting. **D.** HEK293 cells were treated as in (C), and p53 was detected by western blotting. Relative p53 band intensities were quantified by densitometry and the ratios of the p53 to ß-actin are shown (* p <0.05). Wt: FLAG-PICT-1; A: FLAG-PICT-1 (S233A, T289A); D: FLAG-PICT-1 (S233D, T289D).

The translocation of RPL11 from nucleolus to nucleoplasm could be attributed directly to PICT-1 phosphorylation in addition to its degradation. As shown in Figure 7A, significantly less RPL11 protein co-immunoprecipitated with the same amount of PICT-1 protein in UVB-irradiated compared to unexposed control groups. This indicates that DNA damage reduced the ability of PICT-1 to bind RPL11. To determine whether PIKKs participate in this process, the same Co-IP assay was performed following wortmannin or LY294002 pre-treatment. Wortmannin, but not LY294002, prevented UVB radiation from reducing RPL11-PICT-1 binding (Figure 7B). In another experiment, HEK293 cells were transfected with wild-type, PICT-1 (S233A, T289A), or PICT-1 (S233D, T289D) plasmids for 24 h. Cells were then left untreated or were treated with MMC. Cell lysates were immunoprecipitated with an anti-FLAG antibody at multiple time points and RPL11 proteins were detected by western blotting. As shown in Figure 7C, less RPL11 co-immunoprecipitated with wild-type and phosphomimetic PICT-1 than with PICT-1 (S233A, T289A) in MMC-untreated cells. This confirms that sites on PICT-1 phorphorylated by DNA damage regulate its binding with RPL11. MMC treatment did reduce binding between PICT-1 (S233A, T289A) and RPL11 compared to that in MMC-untreated cells; however, this reduction was greater in cells tranfected with wild-type PICT-1 or PICT-1 (S233D, T289D) (Figure 7C). Accordingly, following MMC treatment, cells transfected with PICT-1 (S233A, T289A) had significantly less p53 accumulation than those transfected with wild-type PICT-1 or PICT-1 (S233D, T289D) (Figure 7D).

### PICT-1 regulates DNA damage-induced apoptosis

**Figure 8:**
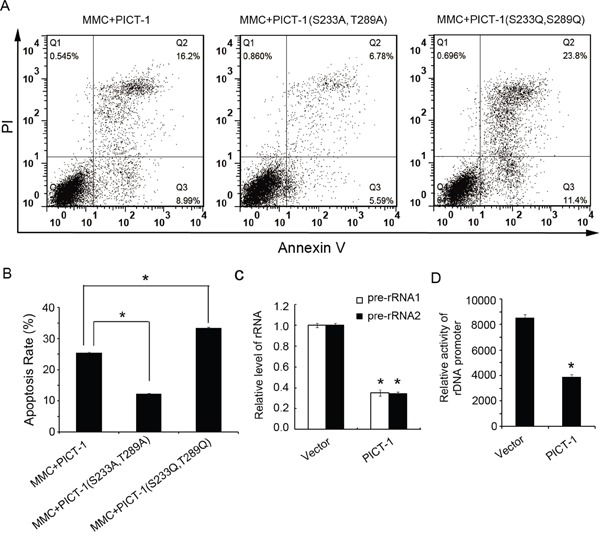
PICT-1 regulates DNA damage-induced apoptosis HEK293 cells were transfected with pFLAG-CMV2-PICT-1, pFLAG-CMV2-PICT-1 (S233A, T289A) or pFLAG-CMV2-PICT-1 (S233D, T289D) for 24 h, and then treated with MMC (10 μg/mL) for another 12 h. Cell apoptosis was detected with the Annexin V/PI staining by flow cytometry. **A.** Representative plots. **B.** Experiments were performed in triplicate, and the data are presented as mean ± SD (*p < 0.05). **C.** HEK293 cells were transfected with pFLAG-CMV2 or pFLAG-CMV2-PICT-1 for 24 h, and then 47S precursor rRNA (pre-rRNA) was detected by qRT-PCR. The experiment was repeated three times and the expression level in control cells was set to 1 (* p <0.05). **D.** pFLAG-CMV2 or pFLAG-CMV2-PICT-1 together with pHrD-IRES-Luc were co-transfected into HEK293 cells for 48 h. Protein concentration was normalized across lysates, and luciferase activity was measured. Data (arbitrary units) from three independent experiments are presented as mean ± SD (* p <0.05).

When DNA is damaged, the DDR can trigger cell cycle arrest and DNA repair or, in the case of irreparable damage, induce p53-dependent apoptosis [45–47]. Therefore, we tested whether DNA damage-induced apoptosis is associated with PICT-1 phosphorylation. HEK293 cells expressing wild-type PICT-1, PICT-1 (S233A, T289A) or PICT-1 (S233D, T289D) were treated with 20 μg/mL of MMC, and apoptosis was detected by flow cytometry. As shown in Figure 8A and 8B, PICT-1 (S233A, T289A)-expressing cells exhibited less MMC-induced apoptosis compared with wild-type PICT-1 and PICT-1 (S233D, T289D)-expressing cells. Moreover, cells expressing PICT-1 (S233D, T289D) had higher rates of apoptosis than wild-type PICT-1-expressing cells following MMC treatment. These results suggest that PICT-1 phosphorylation and degradation plays a role in DNA damage-induced apoptosis.

## DISCUSSION

DNA damage is frequently caused by physical and chemical mutagens, such as UV light, ionizing radiation, and a wide variety of genotoxic agents [48]. It has long been recognized that DNA damage is a major cause of human cancers. To maintain the integrity and fidelity of genomic DNA, cells have evolved a complex signal transduction network that detects DNA damage, then arrests the cell cycle or initiates programmed cell death [34, 35]. Two members of phosphatidylinositol 3-kinase (PI-3) superfamily, ATM and DNA-PK, sense DNA damage and initiate the DNA damage repair response by recruiting and phosphorylating a series of downstream effectors [33]. Recently, several pieces of evidence have linked DNA damage to nucleolus function. First, nucleolar proteome analysis showed that several key proteins involved in the DNA damage and repair pathway are located in the nucleolus [49, 50]. However, their function in the nucleolus following DNA damage is currently unknown. Second, UV or IR radiation can relocate several known nucleolar proteins, such as NPM, nucleolin, Ki67, HRad17 and WRN, to the nucleoplasm [51–55]. Third, DNA damage can affect nucleolar function [56–58]. These findings suggest that crosstalk may exist between DNA damage and nucleolar function, although the molecular mechanism has yet to be revealed. In the present study, we demonstrate that treatment with UV radiation or a genotoxic agent induces marked changes in the staining pattern and levels of PICT-1, a known nucleolar protein (Figure 1). More importantly, two DNA damage response PIKKs, ATM and DNA-PK, co-localize and interact with PICT-1 in nucleoli (Figure 2), and PICT-1 could be phosphorylated by ATM and DNA-PK (Table 1, Figure 3 and 4). Moreover, the DNA damage-induced changes in PICT-1 require PIKKs activation and phosphorylation of PICT-1 residues S233 and T289 (Figure 4 and 5). Thus, PICT-1 may act as an important sensor linking nucleolus stress response and DNA damage. However, what need to mention is that the claim of PICT-1 phosphorylation by ATM is based mainly on several indirect evidences, such as specific phospho-motif antibody of PICT-1 now was not available. Thus, more experiments are needed to confirm PICT-1 as a bona fide target of ATM and DNA-PK.

PICT-1 is an essential ribosome biogenesis factor that is responsible for 5S RNP integration into the ribosome. Depletion of PICT-1 causes RPL11 translocation to the nucleoplasm where it binds and blocks MDM2 activity, allowing p53 accumulation [28, 30]. Therefore, we hypothesized that PICT-1 can participate in nucleolar stress-associated p53 accumulation in response to DNA damage. As expected, we found that both DNA damage-induced phosphorylation and degradation of PICT-1 can cause the movement of RPL11 from nucleolus to nucleoplasm in HEK293 cells. This increased RPL11 interaction with MDM2 and caused p53 accumulation (Figure 6 and Figure 7). Thus, our data suggest that the nucleolar stress-associated increase in p53 is attributable, at least in part, to the PIKKs-dependent phosphorylation and degradation of PICT-1 in response to DNA damage.

The well-known tumor suppressor p53 plays important roles in cell cycle arrest, apoptosis, and maintaining genome integrity [23, 24]. In this study, we also tested whether DNA damage-induced apoptosis is associated with PICT-1 phosphorylation and p53 levels. We found that HEK293 cells expressing unphosphorylatable PICT-1 have lower apoptosis rates than cells expressing wild-type or phosphomimetic PICT-1 (Figure 8A and 8B). These data indicate that DNA damage-induced apoptosis of HEK293 cells was regulated, at least in part, by PICT-1. There are contradictory reports about the function of PICT-1 in cancer. Some studies suggest that PICT-1 is a tumor suppressor, and its over-expression inhibited cell growth and promoted p53-independent apoptosis or death [7, 59]. Nevertheless, some studies report that PICT-1 is oncogenic, sequestering RPL11 in the nucleolus to repress MDM2 [10, 11, 30]. In this study, we showed that PICT-1 over-expression inhibited transcription of the rDNA gene (Figure 8C and 8D). Previous studies have reported that the transcriptional inhibition of rDNA can induce p53-independent cell death or apoptosis. Therefore, we speculate that the DNA damage-induced “PICT-1-RPL11-MDM2” p53-dependent apoptosis is different from the death mechanism induced by PICT-1 over-expression.

**Figure 9:**
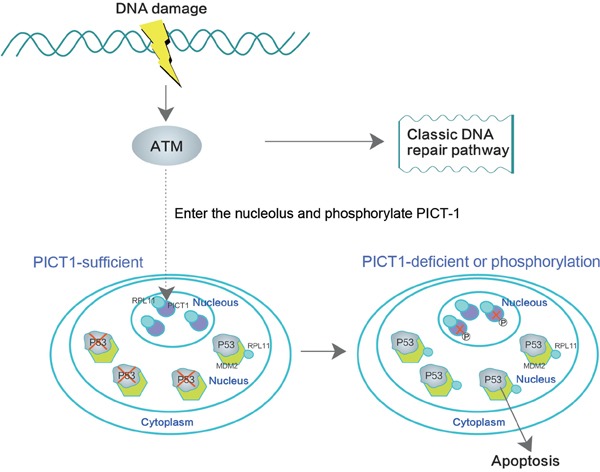
PICT-1 is a key regulator of the DNA damage-induced MDM2-p53 signaling pathway ATM binds and phosphorylates PICT-1 in the nucleolus upon DNA damage, and PICT-1 phosphorylation at S233 and T289 is responsible for DNA damage-induced reduction in PICT-1 protein. Both PICT-1 phosphorylation and degradation release RPL11 from the nucleolus to the nucleoplasm, where it binds and blocks MDM2 activity. This allows p53 accumulation and induces apoptosis.

In summary, our study demonstrates that, upon DNA damage, ATM can bind to and may phosphorylate PICT-1 at S233 and T289 in nucleoli. Phosphorylation at these sites then induces redistribution and degradation of PICT-1. PICT-1 phosphorylation and degradation release RPL11 from the nucleolus to the nucleoplasm. RPL11 then binds MDM2, allowing p53 accumulation. We conclude that PICT-1 is a major nucleolar sensor of DDR and the important upstream regulator of p53 activation via RPL11-MDM2-p53 pathway (Figure 9).

## MATERIALS AND METHODS

### Cell lines, plasmids and reagents

HEK293 and U251 cells were purchased from the American Type Culture Collection (ATCC) and maintained at 37°C with 5% CO_2_ in Dulbecco's modified Eagle's medium (DMEM) supplemented with 10% fetal bovine serum. Human wild-type PICT-1, PICT-1 (S233A, T289A), PICT-1 (S233A), PICT-1 (T289A), PICT-1 (S233D, T289D), PICT-1 (S233D), PICT-1 (T289D) and RPL11 were amplified by PCR and subcloned into pFLAG-CMV2, PEGFP-C1 or DsRedC1 plasmids (Clontech, Palo Alto, CA), respectively. The pHrD-IRES-Luc plasmid containing the promoter sequence for the rDNA gene was a kind gift from professors Ke Y and Jacob ST [60, 61]. ATM shRNAs were from TRC shRNA librariy, Sigma-Aldrich (St. Louis, MO, USA). Anti-PICT-1, anti-p53, anti-β-actin, anti-FLAG, anti-phospho-DNA-PKcs (Thr 2609), anti-DNA-PKcs and anti-nucleolin antibodies were purchased from Santa Cruz Biotechnology (Santa Cruz, CA, USA). Anti-ATM antibody was from Novus (Littleton, CO, USA). Anti-phospho-ATM (Ser1981) and anti-PICT-1 antibodies were from Cell Signalling (Beverly, MA, USA). Anti-RPL11 and anti-MDM2 antibodies were from Abcam (Abcam, Cambridge, UK). Anti-phospho-(Ser/Thr/Tyr) substrate antibody was from Abnova. HRP- and fluorescein-labelled secondary antibodies and ECL were obtained from KPL (Gaithersburg, MD, USA). LY294002, wortmannin, KU55933, NU7026, MG132 and CA-074 were obtained from Sigma-Aldrich. Propidium iodide (PI) was purchased from Sigma-Aldrich. The Alexa® Fluor 488 Annexin V/PI Dead Cell Apoptosis Kit was from Invitrogen (Carlsbad, CA, USA). MMC was from Sigma-Aldrich.

### Drugs and UV treatment

U251 or HEK293 cells left untreated or were pre-treated with 100 nM wortmannin (a broad inhibitor of PIKKs), 5 μM LY294002 (a lipid phosphatidylinositol 3-kinase inhibitor), 10 μM KU55933 (a specific ATM inhibitor) or 10 μM NU7026 (a DNA-PK-specific inhibitor) for 30 min [42, 62, 63], and then exposed to UVB light (10 J/m^2^) or MMC (10 μg/mL). Cells were analyzed using western blotting or confocal microscopy at the indicated time points.

### Western blotting

Proteins separated by SDS-PAGE were transferred to PVDF membranes. Membranes were blocked with 5% non-fat milk (2 h at room temperature) and then incubated with the desired primary antibodies overnight at 4°C. After a 4 h incubation at room temperature with an HRP-coupled secondary antibody, the proteins were detected using ECL.

### Nucleolus fractionation

Nucleoli were isolated from HEK293 cells as previously described [42]. Nuclear and nucleolar fractions were analyzed by western blotting using the indicated antibodies.

### Confocal microscopy

Cells were seeded on coverslips and treated with UVB light or MMC in the presence or absence of inhibitors. Cells were fixed with 4% paraformaldehyde for 15 min at room temperature, then permeabilized with 0.1% TritonX-100 for 15 min. Cells were blocked with 1% BSA for 3 h at room temperature, then incubated with primary antibodies overnight at 4°C. Cells were stained using fluorescein-conjugated secondary antibodies and 0.5 μg/mL DAPI, then imaged using a confocal microscope.

### Immunoprecipitation

HEK293 cells were dispersed by sonication in 500 μL immunoprecipitation buffer (20 mM Tris-HCl (pH 7.4), 150 mM NaCl, 1 mM EDTA, 1mM EGTA, 1% TritonX-100, 2.5 mM Na_4_P_2_O_7_, 1 mM β-glycerophosphate, 1 mM Na_3_VO_4_ and 1 mM PMSF). After removing cell debris by centrifugation, the supernatant was incubated with the indicated primary antibodies and protein A/G agarose or anti-FLAG M2 affinity gel (Sigma–Aldrich) for 4 h at 4°C. Beads were collected and washed three times with IP buffer. The resolved proteins were immunoblotted with the indicated antibodies.

### Apoptosis assay

HEK293 cells were transfected with pFLAG-CMV2-PICT-1 (wt), pFLAG-CMV2-PICT-1 (S233A, S289A) or pFLAG-CMV2-PICT-1 (S233D, S289D) for 24 h, then treated with MMC (10 μg/mL) for 12 h. Cells were dissociated with EDTA-free trypsin and stained with annexin V and PI according to the manufacturer's protocol (Invitrogen, V13241). Apoptosis was quantified by flow cytometry (BD Science), with annexin V-positive cells considered apoptotic.

### Real-time quantitative PCR (qPCR) for pre-rRNA expression

Total RNA from HEK293 cells transfected with pFLAG-CMV2 or pFLAG-CMV2-PICT-1 was extracted using TRIzol (Invitrogen Corp., Carlsbad, CA, USA). First-strand cDNA synthesized by random primers was submitted to qPCR amplification using SYBR Green. Primers for pre-rRNA detection used in qPCR are as described previously [64, 65].

### Luciferase reporter assay

To evaluate the effects of PICT-1 over-expression on rDNA promoter activity, HEK293 cells were transfected with control plasmid or pFLAG-CMV2-PICT-1 together with pHrD-IRES-Luc for 48 h. The luciferase activity of cell lysates was measured using a luciferase assay kit (Promega, Madison, WI, USA).

## Abbreviations

ATM: Ataxia-telangiectasia mutated
Co-IP: co-immunoprecipitation
DDR: DNA damage response
DNA-PK: DNA-dependent protein kinase
GLTSCR2: glioma tumor suppressor candidate region gene 2
LOH: loss of heterozygosity
MDM2: murine double minute 2
MMC: Mitomycin C
PICT-1: protein interacting with carboxyl terminus 1
PI3K: class I phosphoinositide 3-kinase
PTEN: phosphatase and tensin homolog
PIKKs: phosphatidylinositol 3-kinase-like kinases
qRT-PCR: real-time quantitative PCR.
